# The Lost Social Context of Recovery Psychiatrization of a Social Process

**DOI:** 10.3389/fsoc.2022.832201

**Published:** 2022-04-04

**Authors:** Alain Topor, Tore Dag Boe, Inger Beate Larsen

**Affiliations:** ^1^Department of Social Work, Stockholm University, Stockholm, Sweden; ^2^Faculty of Health and Sports Sciences, University of Agder, Kristiansand, Norway

**Keywords:** recovery, mental, individual, social, cultural, relationships, societal, psychiatrization

## Abstract

From being a concept questioning the core of psychiatric knowledge and practice, recovery has been adopted as a guiding vison for mental health policy and practice by different local, national, and international organizations. The aim of this article is to contextualize the different understandings of recovery and its psychiatrization through the emergence of an individualizing and de-contextualized definition which have gained a dominant position. It ends with an attempt to formulate a new definition of recovery which integrates people in their social context. Research results from various follow-up studies showing the possibility of recovery from severe mental distress have stressed the importance of societal, social and relational factors as well of the person's own agency when facing their distress and reactions from their environment. These researches were published in the 1970s and 80s; a period of struggle for liberation from colonialism, of struggle by women and black people for their civil rights, and a time of de-institutionalization of services directed toward the poor, elderly, handicapped, prisoners, and people with mental health problems. Recovery research pointed at the central role of individuals in their recovery journey and it was understood as a personal process in a social context. However, with neo-liberal political agenda, the personal role of individuals and their own responsibility for their well-being was stressed, and contextual understandings and the role of social, material and cultural changes to promote recovery faded away. Thus, during recent decades recovery has been mostly defined as an individualistic journey of changing the persons and their perception of their situation, but not of changing this situation. Contextual aspects are almost absent. The most quoted definition accepts the limits posed by an illness-based model. This kind of definition might be a reason for the wide acceptance of a phenomenon that was initially experienced as a break with the bio-medical paradigm. Recently, this dominant individualized understanding of recovery has been criticized by service users, clinicians and researchers, making possible a redefinition of recovery as a social process in material and cultural contexts.

…ideologies can be liberating while they are still in formation and oppressive once they become institutionalized (Scheper-Hugues and Lowell, 1986, p. 162).

## The Social Character of Mental Health

The topic of this article could be seen as paradoxical. How could recovery be part of the psychiatrization of society? From the beginning recovery was perceived as a process of leaving mental health problems and services behind and to be ≪recovered≫ thanks to changes in one's social and material conditions. It was about de-psychiatrization of one's life. However, recovery became psychiatrized and professionalized and transformed in a never-ending process of being ≪in recovery≫. People with mental health problems were again defined as suffering from diseases and as in need of psychiatric interventions mostly in “recovery-oriented” services.

Recently the most common understandings and definitions of recovery (Anthony, 1993; Leamy et al., 2011) have been criticized for their individualistic and normative aspects (Harper and Speed, 2014; Karadzhov, 2021; https://recoveryinthebin.org), as they do not mention the importance of social living conditions such as financial resources, housing and general living conditions (Harper et al., 2015). The critics have highlighted the social character of mental health problems and recovery, but also of the mental health field itself (Topor et al., 2011; Tew et al., 2012; Rose, 2018; Rose and Kalathil, 2019; Karadzhov, 2021). Materialities (Larsen et al., 2021), places (Duff, 2012; Doroud et al., 2018), and social relations (Topor et al., 2016a; Price-Robertson et al., 2017) have also been stressed as missing in recovery research. Finally, the narrative/biographical character of dominant recovery research putting recovery into the formula of a personal, chronological, order, at the cost of social aspects, has been questioned (Bøe et al., 2021).

Thus, it seems that people in recovery studies are floating in a social vacuum where their possibilities and capacities to live a satisfying life mostly depend on themselves and their own efforts. People in many recovery studies are not connected to their living conditions in a time of growing inequalities, social welfare cutbacks, and deteriorating conditions on the labor market. The lived life and thereby the basis for people's sense of self are excluded from our knowledge about recovery.

## Aim and Methodological Considerations

In this article we aim to place the development of medicalized, psychologized, and individualistic definitions of recovery in their contemporary historical contexts. Here we might see a special case of psychiatrization (Beeker et al., 2021). Not only that social, material and cultural conditions for people's life behind mental distresses are rendered invisible, but also the same conditions' central role for improvements and positive changes regarding these distresses. Finally, we propose the starting point for a contextual definition of recovery. As Ramon (2018) writes “… it is important to include reflections of recovery journeys alongside formal research” (p. 2).

Beeker et al. (2021) point out that psychiatrization is hard to define because of the diversity found in psychiatry itself. However, they suggest the following working definition:

“[P]sychiatrization [is] a complex process of interaction between individuals, society, and psychiatry through which psychiatric institutions, knowledge, and practices affect an increasing number of people, shape more and more areas of life, and further psychiatry's importance in society as a whole” (p. 3).

In this paper we explore the emergence of recovery in light of these aspects, but in a period of de-institutionalization and de-psychiatrization. We also explore recovery's later transformation to a psychiatric and psychiatrizing concept and the context of this radical change. Finally, we point at discontents with the psychiatrization of society and of recovery and suggest a de-psychiatrizing definition of recovery.

### Methodological Considerations—Some Words About Words

Goffman (1976/1979) made once a methodological statement:

The particular matters I want to consider raise three distinct and general methodological questions that should not be confused: discovery, presentation, and proof. Only the first two will here be at issue… (24).

We adhere to Goffman's distinction and hope the reader will bear it in mind when reading this article.

The reader should also keep in mind that when writing about psychiatric diagnosis and recovery one is confronted with important questions regarding both language and technical issues (Boyle, 2015; Topor et al., 2018). The definition of a central diagnosis like schizophrenia varies with time and place. Boyle (2002) argues convincingly that the dementia praecox of Kraepelin might have been a completely different illness than the schizophrenia psychiatrists diagnose today (Hegarty et al., 1994). Recovery has also been analyzed and defined in many ways (clinical, total, social, personal, relational, etc.), based on a range of criteria (Davidson and Roe, 2007; Slade, 2009). Where possible, we have presented short *ad hoc* definitions. Throughout the paper, we do not use “illness,” but rather “problems” and “distress.”

Last but not least, who are we talking primarily about? All the terms in use, such as *patient, client, consumer, user, citizen*, and *survivor* are imbued with different ideological and scientific positionings. Therefore, we will refer to persons in inpatient settings as “patients” and outside these settings as “persons” or “people,” “person with a diagnosis of severe mental problems” or “service users.”

Follow-up studies have been criticized because of unclear definitions of central concepts and because of technical problems often inherent to this kind of study (biased population, drop-outs, different outcome measures…). The studies we present have also faced such criticism; however, they have been published in peer-review journals and the interested readers are kindly asked to maintain a critical stance toward both the referred literature and how this article's authors have used it for their own purposes.

## The (de-)Construction of Chronic Mental Illnesses

A re-occurring paradox in psychiatry is the simultaneous claims about discoveries of constantly more efficient treatment interventions on the one hand and the definition of most diagnosis/illnesses as “chronic,” or “long-term” on the other (Warner, 2004; Priebe et al., 2013).

Specifically, schizophrenia is referred to as a progressive destruction of what constitutes a human being (Kraepelin, 1919/1971; Frith and Johnstone, 2003). Ey (1977), a major French psychiatrist summarized the different definitions of schizophrenia as:

The loss of entity, that constitutes the individual, regression into delusions, detachment from reality, disturbance in communication are all various aspects of the emergence of a person without person and of a world without world, which is the very essence of schizophrenia (p. 64).

Therefore, recovery from schizophrenia has seldom been mentioned in literature as a consequence of a specific intervention, if it ever occurred. Kraepelin routinely considered patients improving as misdiagnosed (Harding et al., 1987c). Bleuler (1911/1950) stated: “*As yet I have never released a schizophrenic in whom I could not still see distinct signs of the disease…”* (p. 256, italics in the original). The history of schizophrenia is paved with attempts to maintain the chronic character of the diagnosis, so to explain the occurrence of recoveries Langfeldt (1937) created a special diagnosis “schizophreniform psychosis,” a disease like schizophrenia in every aspect, except that the person recovered. However, remission, a time-limited recovery, could be accepted as a possible stage in the “natural course” of the general decline of the person.

The depressive character of psychiatric thought might, at least partly, have been a consequence of “the clinician illusion” (Cohen and Cohen, 1984), as many psychiatrists developing classifications were working in total institutions. There, they met persons with a diagnosis of severe mental problems when they were ill. Discharged patients who did not “relapse” disappeared from their sight, thus creating a biased experience-based body of knowledge about mental health problems as chronic illnesses (See also Bleuler, 1978).

Over the years different interventions were developed and presented as successful, such as ETC, psychosurgery, therapeutic community, the first and second-generation neuroleptics and the atypical one etc. Nevertheless, recovery from schizophrenia was not on the agenda. In the fourth edition of DSM (American Psychiatric Association, 2000) it was stated that: “Complete remission (i.e., a return to full premorbid functioning) is probably not common in this disorder.” (p 282)

This definition constitutes an established but odd way to measure an improvement, as if recovery was about a kind of return journey in one's history. As if a person who had experienced the distress of severe mental illness and the challenges connected to stigma and to mental health care could or would return to a premorbid state (the state that might have triggered the problems). This reflects a central psychiatrizing pattern where the illness exists separate from and independently of life events and experiences; a figure we will come back to.

### Recovery as a Probable Outcome

The publication around 1980 of several follow-up studies of persons diagnosed with schizophrenia constituted a challenge to the dominant medical understanding of severe mental illnesses. Living conditions and life-events, often connected to these conditions, played a central part in peoples mental health. This knowledge became part of a process of de-psychiatrization in western societies connected to the radical de-institutionalizing movement and focusing not only on psychiatric total institutions, but also on psychiatry's and psychiatrists' power-based knowledges and practices (Foucault, 1980; Scheper-Hugues and Lowell, 1986).

Thus, when the WHO (1979) started to publish the results from its international follow-up study they were met with skepticism and rejection. A first article on the US results was sent back to its authors, the reviewers arguing that they had to reconsider their statistics (John Strauss, personal communication). The proportion of persons in recovery was far too high to be plausible.

The WHO study was not critical to traditional psychiatric knowledge only because of the high percentage of recovered persons it showed. An even more unacceptable result was that the proportion of recovered persons was higher in low-income countries, with a limited presence of medical mental health resources, compared to high-income countries (Hopper et al., 2007; Mills, 2014).

However, the WHO study was followed by other studies presenting results showing that recovery from schizophrenia was not only a possibility, but that (in most studies) about one-fifth to one-third of the persons diagnosed with schizophrenia showed complete recovery (Bleuler, 1978; Ciompi, 1980; Harding et al., 1987a,b; Warner, 2004). This meant that they did not present any symptoms of the illness and were living independently in the community. Around the same number were classified as socially recovered as they could show mild but not invalidating symptoms and lived in the community, although with some support.

Harding et al. (1987a,b) published a follow-up study of patients from a mental hospital that were not able to be discharged when the first-generation neuroleptics had been administered to all the patients in the actual institution. At follow-up, 30 years later, 68% did not display any sign of schizophrenia, and 50% were not using neuroleptics.

Warner (2004) published a compilation of recovery studies conducted through the twentieth century. His review showed a total recovery rate fluctuating between 10 and 20 percent and a social recovery rate between 30 and 40 percent over the century. He also found that the general use of first-generation neuroleptics in the 1950s did not improve recovery rates.

Bleuler (1978), the son of Eugen Bleuler who coined the term “schizophrenia,” published the results of a follow-up study that had lasted over 30 years. Unlike his father he stayed in touch for decades with patients even after they were released from hospital, and included them in his study. His results showed that 23% were fully recovered and a further 43% were socially recovered. Father and son's different appreciations of the possibility of recovery might be seen a dramatic illustration of the “clinician's' illusion” thesis.

### Recovery's Social Context

Besides showing that recovery was not rare, these studies showed that recovery could not be connected to specific treatment interventions. The consequences of the “psychopharmacological revolution” could not be detected in these studies. Recovery occurred at about the same rate at different times when different interventions were the golden rule of the day.

Different hypotheses were formulated to explain these results. Warner (2004) made statistical calculations to study possible reasons for the greatly reduced probability for recovery during a period between 1921 and 1940. His conclusion pointed at the increased un-employment rates during the Great Depression before World War 2 as the most probable explanation.

Regarding Harding et al.'s results (Harding et al., 1987a,b), we know that the patients remaining in the mental hospital after all in-patients had received first-generation neuroleptic treatment were offered a long-term rehabilitation program. Thereafter, patients began to leave the hospital after only a few months. Coming out, they were offered a range of residence alternatives and on-going rehabilitative support in the community. Analyzing these data, DeSisto et al. (1995) stressed the importance of hope, relational continuity, and collaboration between users and professionals for sustained recovery.

Different hypotheses regarding the difference between high- and low-income countries recovery rates (WHO, 1979) were formulated (Warner, 2004; Mills, 2014). Some were about the permeability of the work market in the latter, making it easier for people to find a workload appropriate to their actual condition, and about the presence of extended families in low-income countries spreading the family burden among several members, thus easing it for each of them. Another hypothesis was about the local systems of beliefs about the causes of madness. Religious or spiritual explanations were considered as more frequent in low-income countries and were supposed to have less severe consequences for the people and their networks' readiness to deal with problems. In contrast to medical expertise-based interventions, spiritual understandings could leave a greater possibility for people to act against the problems and thus to keep a hopeful mood (Waxler, 1979). Finally, a provocative hypothesis is that the higher recovery rate in low-income countries could be caused by the scarcity of bio-medical interventions and hospitalization possibilities. People in crisis would get Western medical treatment and be medicated, but as soon as the crisis was over, they would return to their villages far from the places offering medical interventions. As an unattended consequence, they would avoid long-term medication and its problems (see also Moncrieff, 2009; Harrow et al., 2012; Mills, 2014). However, one should not negate the existence of ill-treatment of persons with severe mental health problems in low-income countries.

It is noteworthy that these explanations were all basically social/societal and became part of a global questioning of the medical, psychiatric, framework. Thus, they put new questions on the research agenda about the conditions for favorable recovery journeys (Mezzina et al., 2006).

## De-Institutionalization

In fact, these early follow-up studies results were threatening the vision of mental illnesses as illnesses and helpful interventions as medical interventions. They could be seen as a part in a spirit of this time of de-psychiatrizing mental health. If recovery could not be connected to specific medical interventions and if social, cultural, and societal factors were determinant for a recovery process, then mental illnesses were not illnesses. The knowledge collected about recovery and its conditions and practices [“Le savoir des gens” – “Peoples' knowledge,” as Foucault (1980) mentioned] became part of the de-psychiatrization of society.

It seems probable that the de-institutionalization of psychiatry had its roots in a global liberation struggle. The publication of the above-mentioned studies coincides and interacts with societal circumstances. The post-World War 2 period was characterized by a wide range of liberation movements and struggles for applied citizenship. People from colonized countries participated in World War 2 on the side of their colonial powers and this fueled their struggle to be recognized as independent countries. These struggles influenced both the people fighting for their independence and people in the colonial powers and other high-income countries. Struggles for dignity and liberation in Europe and North America came to include basic civil rights, both for women and ethnic minorities (Davidson et al., 2010), but also for homosexuals (Kirk and Kutchins, 1992). Beeker et al. (2021) mentioned “…the de-pathologization of homosexuality and its removal from DSM-II in 1973” as “the most prominent case of de-psychiatrization” (p. 3). But one should not forget that both women and racialized people have been and still are psychiatrized and that their liberation struggle is at least partly about the psychiatrized boundaries of normality (Read and Beavan, 2013; Read et al., 2013).

Another struggle front was about developing welfare states and guaranteeing coverage of basic needs in the case of unemployment, sickness, and poverty. Finally, a process of de-institutionalization was initiated regarding the elderly, the handicapped, prisoners and mental health patients. These groups often lacked basic civil rights and the above-mentioned struggles have to be understood in the light of the cold war, where Western Europe and the US criticized the Soviet Union for the lack of democratic rights.

Already in the 1940s, long before the first-generation neuroleptics came into use, the number of in-patients in some states in the US diminished (Scull, 1984). After World War 2 this became the dominant trend in many high-income countries' psychiatric care. The closing of mental hospitals has been associated with the use of the first-generation neuroleptic drugs. However, as we mentioned, it started long before neuroleptics came in use, but in countries such as Sweden and Italy, the downsizing of mental hospitals waited until the end of the 60s, long after the use of these drugs had become generalized (Markström et al., 2004; Carta et al., 2020). De-institutionalization has sometimes been reduced to de-hospitalization; a mere closing of inpatient institutions. These situations resulted in homelessness and abandonment, leading to extensive tragedies (Scheper-Hugues and Lowell, 1986; Dear and Wolch, 1987).

De-institutionalization was not only to be understood as the closing of the total institutions, but also as the construction of alternatives in the community to facilitate the inclusion of the former segregated groups in society and the challenging of medicalized knowledge and practices about madness (Scheper-Hugues and Lowell, 1986; Rotelli, 1994; Carta et al., 2020).

This move from a specialized and segregated field open only for experts by profession to the public agenda including societal and political discussions about madness, mental health treatments and service organizations could be both illustrated and pushed forward by the publication in the 1960's first 2 years of Foucault's Histoire de la folie á l'age classique (1961), Goffman's Asylums - Essays on the social situation of mental patients and other inmates (1961), Szasz's The myth of mental illness (Szasz, 1961) and Liang's The divided self (Laing, 1960). From different perspectives they all considered psychiatry as a social field and mental illness as a medical construction that could and should be de-constructed.

De-institutionalization, de-psychiatrization and psychiatrization are complex concepts and their practice should not be reduced to simple processes. Already in Castel (1976), Castel pointed in “The psychiatric order” at modern psychiatry's new challenge. When the number of diagnosed persons was limited, the maintenance of order in society could be organized through the exclusion of the deviant in total institutions. When the number of deviants grew, exclusion threatened the very base of society; the production of goods. In this context the mission of psychiatry changed, from taking care of “lunatics” to taking care of the population. Psychiatry had to find solutions to maintain “people at risk” in the society and first of all as work force. Castel argues that the deployment of psychiatric structures in the society opened for constant monitoring and for the use of new techniques like behavioral therapy, but also of “constant performance evaluation and assessment from birth to death” (p. 290. See also “The advanced psychiatric society—the American model”; Castel et al., 1979).

However, another result of the closing of mental hospitals was the presence in the community of a growing number of persons with their own experience of mental distress and of psychiatric care, persons whose words and experiences could now be heard publicly without being immediately interpreted by mental health professionals as symptoms of their illnesses (Chamberlin, 1978; Deegan, 1988; Romme et al., 2009).

### The De-Psychiatrization of the “Patient”—The Discovery of the Patient as a Person

De-psychiatrization of society also touched our notion of the mad person. Once the possibility of recovery from “illnesses” previously considered as “life-long” was established, studies focused on what benefits a recovery journey? What might hinder it?

At that time, the voices and experiences of service users had gained a certain credibility. Earlier narratives from users were mostly silenced or interpreted through the lenses of the experts by profession (Freud, 1905/1997). This increased credibility might be considered as one of the major contributions of the growing independent service users' movements and of recovery research; the transformation of the patients diagnosed as out of their mind and of reality, reduced to their symptoms, “a person without a person” into an expert with experience-based knowledge. The “discovery” of the patient as a person separated and not reduced to a diagnosis was reflected in titles of publications at that time, such as “The patient with schizophrenia as a person” (Strauss, 1994) and “From the mental patient to the person” (Barham and Hayward, 1991).

A central part of these narratives was the discovery of the importance for treatment results of professionals' confirmation of the patient as a person (Denhov and Topor, 2012; Topor and Denhov, 2015). Re-occurring concepts in the studies concern seeing “the service user as an individual, not just a patient” (Farrelly and Lester, 2014); as more than just a “…number, diagnosis, or set of diagnoses…” (Shattell et al., 2007). The basic aspect of it is the confirming of the user's “share humanity” (Sandhu et al., 2015) and as a “whole human being” (Grim et al., 2019) and “a fellow human being,” “putting the psychosis in brackets and cultivating all that is healthy” (Bjornestrand et al., 2018). Interpersonal aspects are focused on in terms of the “inviting attentiveness” on the part of the professional, which offers the user a “vitalizing space” (Topor et al., 2014; Ljungberg et al., 2015).

These studies have given us important contributions to our understanding of the recovery journeys and of hindering and contributing factors. They are mostly about the person's own efforts and struggle. About the person's development of different ways to deal with their environment, their families, friends, professionals, and the vicissitudes of everyday life and of what was and often still are considered as symptoms (Deegan, 1988; Romme and Escher, 1989; Davidson et al., 2006; Topor et al., 2016b).

This relational aspect of de-constructing the patient and re- and co-constructing the person (Price-Robertson et al., 2017) constituted a challenge to traditional knowledge about the illnesses that these persons were said to be affected by, attacking their capacity for and interest in social relationships (Frith and Johnstone, 2003). The patient remains a person but hidden by the clinical gaze in different institutions, characterized by their loss of power. The professional has to break with a strict clinical worldview to re-establish the patient/client as a person in their own eyes, and as a partner in a possible joint venture.

### Experience, Narrative, and Knowledge

The liberation of users' voices was of central democratic importance. However, these voices were sometimes given a special status, replacing what Foucault (1961/1972) called the monolog of reason (the psychiatrists) about madness (the patients) by a counter monolog as users were declared to be the experts about their own situation and thus, as the new experts, that could not be criticized (Bøe et al., 2021). However, accepting the patient as a person means accepting their storied experiences as everybody else's narratives and not as symptoms of illness to be interpreted by the holders of a special knowledge. Personal narratives might play different roles in everyday, therapeutic, political and research settings.

On an everyday, personal level the function of developing an experience-based narrative might be to create a personal understanding of one's history and could be of central importance in a recovery process (Boyle and Johnstone, 2018). The problem with the use of these narratives, as well as of professionals' narratives, in research is that the knowledges produced in both cases are the product of immediate individual experiences and the person's agenda. Complexity and contradictions are lost. They are also, like all narratives, influenced by dominating, master narratives (Hydén, 1995) about how a story should be told and what elements are accepted in different contexts (Llewellyn-Beardsley et al., 2020). In a period of psychiatrization of social processes and of our understanding of ourselves and our lives, a looping effect can be noticed, where persons accept and reproduce the definition of themselves as mentally ill and in need of a diagnosis and medication (Hacking, 1998; Mills, 2015). Personal narratives reflect important experiences but run the risk of missing the contributions and contradictions from contextual and social analysis of their conditions (Bøe et al., 2019). This opens for the possibility to analyze the psychiatrization of society as both a top-down and also a bottom-up process.

Thus, as important as individual narratives might be, they should be critically scrutinized to protect knowledge from what Bourdieu (1986) called the biographical illusion, where the chaos of life is ordered through a co-creative process between the person and the researcher; a constructed order where earlier events tend to be presented as causes of later developments. Definitions where recovery is seen as individual stories along a *temporal* trajectory lose from sight complexity and the social aspects of *spatial* interaction (Larsen et al., 2021).

Citizenship includes the right to tell one's story but also to be part of conflictual dialogs, replacing the monolog of Power/Reason. This could be seen as another aspect of the de-psychiatrization of mental illness and society.

## From The Social Person to The Isolated Individual—The Psychiatrization of Recovery

If psychiatrization is defined as “a complex process of interaction between individuals, society and psychiatry…” (Beeker et al., 2021, p. 3) it is of core importance to study not only the impact of psychiatry on society, but how these three actors influence each other. The discovery of the probability of recovery from severe mental illnesses, of the importance of social, cultural and societal aspects and of the patient as a person and an agent coincides with a period of global political changes. De-institutionalization and de-psychiatrization (“anti-psychiatry” (Castel, 1981) and “Alternatives to psychiatry” (Collectif International, 1977) started at the end of the thirty years following World War 2 characterized, as we have seen, by different liberation movements and the construction of the modern welfare state. It also started at the beginning of a neo-liberal period in world economy, politics and understanding of human beings.

The medicalization of society, psychiatry and recovery can be understood as a result of tendencies in different fields during the ultimate decades of the twentieth century.

Regarding society it is about a neo-liberal shift that was expressed by different heads of states and concretized in political decisions jeopardizing the welfare states in different countries. In 1987, Margaret Thatcher, UK prime minister, declared that there was “no such thing as society, just men and women…” The same year, Ronald Reagan started his first presidency by putting an end to federal help to develop community mental health services.

Regarding *psychiatry* the DSM III (APA, 1980) was launched as psychiatry's return to real science and the first step to develop a scientific base to the proposed diagnosis and thus to be the base for the development of adequate treatments. It was followed by the arrival of the second-generation anti-depressant and anti-psychotic medicines with promises of high efficiency, finally proving that mental distress were illnesses like all other illnesses and psychiatry a branch of medicine. It is a paradox that despite these success stories the American Psychiatric Association (1994) continued to declare schizophrenia a chronic disorder.

The neo-liberal changes in policy and the medicalization of psychiatry became *a joint venture* with US president Georg Bush proclaiming the nineties as “The Decade of the Brain” (later to become “The century of the brain”). While the next president, Bill Clinton, 1996, declared that ≪The era of the big state is over…≫ and cut down different forms of social security systems (Wacquant, 2009). State money was instead dedicated to research about the biological causes of mental illnesses and to information to the public about mental illnesses that could be cured thanks to new medicines.

Regarding *the individuals*, neo-liberal ideology invaded policies and thereby our understanding of human beings and social problems (Evans-Campbell et al., 2006; Frances, 2013; Ramon, 2018). Public support for poor, discriminated and fragile persons was reduced and the single person's responsibility and capacity to control their own life was stressed (Wacquant, 2009). The ideal of the independent, self-made (wo)man became dominant (Cushman, 1995; Pearlin et al., 2006). Welfare discourses and agencies became penetrated by workfare. Policies where people are guaranteed support if in need were replaced by workfare's conditional aid where the same person first had to prove their willingness to work and to manage themselves before eventually getting some subsidies (Peck, 2001).

However, at the same time having a psychiatric diagnosis became the main road for poor people to get some support. This psychiatrization of society is reflected in diverse, sometimes contradictory numbers. Such a contradiction is about welfare state interventions. At the same time these interventions were under attack, the number of persons receiving monetary support because of mental illness increased, from 1.25 million adult American citizens in 1987 to 4,2 million. Twenty-five years later (Whitaker and Cosgrove, 2015). In schools disturbing kids had to be transformed into disturbed kids to get any help one has to be diagnosed. Recovering, or at least claiming to be totally recovered, entails the risk to lose ones' means of subsistence.

Since the publication of the DSM-I we have witnessed an increased number of diagnoses. In this first edition, 1952, there were 152 distinct disorders. In 1984, with the publication of the third edition, there were 228 diagnoses and finally, in the present fifth edition, there are 541. Parallelly with this inflation of diagnoses a growing number of persons (adults and children) have been declared to present symptoms of mental illness. After the publication of DSM IV one third of the adult US population were considered to have a mental illness. Disturbing kids had to be transformed into disturbed kids. One of seven (age 8–15) were said to show symptoms corresponding to mental illness. This was connected to a huge increase in public spendings for psycho-pharmacological treatment; In the US, from 870 million dollars in 1987 to 35 billion, 2010 (Whitaker and Cosgrove, 2015, p 117). This development was mirrored in other high-income countries.

These numbers point at a dialectical relation between government policies (top-down) and citizens expressed needs (bottom-up). If the dominant ideology about social conflicts and their consequences is medical and if a diagnosis is the key to be helped, then people will reclaim diagnosis and interpret their problems and the distress connected to them as signs/symptoms of an illness inside of them. They will identify themselves as ill and claim for adequate diagnosis and, of course, for what is presented as high-quality, scientifically evidence-based interventions; medicine. The state will give priority to services quickly diagnosing their “consumers” and prompt to offer them such interventions. This self-reinforcing system is good at avoiding critical reviews as all the involved parties find at least short-term gains, even if resistance against psychiatrization can be noticed (Beeker et al., 2020).

This individualization and “responsabilization” came to affect our understanding of recovery and what kind of support people needed (Rose, 2014). However, few recovery studies problematized these changes; instead, most integrated an individualized and de-contextualized perspective (Duff, 2012; Karadzhov, 2021). The newly discovered capacities of persons with a diagnosis of severe mental problems were turned against them and used as an argument to reduce the supporting interventions directed to them in order not to create what were defined as negative dependencies instead of interconnectivity, solidarity and brother/sisterhood and the user as a consumer in a free market (Mills, 2014; Rose, 2014; Woods et al., 2019).

Thus, it might be considered as a paradox that the possibility of recovery came up on the agenda, at least in the US, while people with mental health problems were often dumped and abandoned in the streets to homelessness (Dear and Wolch, 1987; Knowles, 2000); at a time when new concepts were coined such as “the new chronic patients” with no place to stay (Scheper-Hugues and Lowell, 1986, p. 176).

At this point, the psychiatrization of society, transforming poor people into ill people, became connected to the psychiatrization of recovery.

### Recovery as a Never-Ending Personal Journey

In summary, the discovery of the patient as a person, as an agent, and the ideology of a strong independent self together with the neo-liberal discourse about the individual's responsibility for their own fate created and reflected a new spirit. This spirit was distant from the original social, material and contextual understanding of people's recoveries in the complexities of lived life and construction of a more satisfying sense of self. Illnesses had to be treated medically and individually. Additional supports should then be directed to improving the person's understanding of their situation, and not to their factual situation, to avoid dependency.

This new spirit found its ultimate formulation in the most quoted definition of recovery (Anthony, 1993):

Recovery is a deeply personal, unique process of changing one's attitudes, values, feelings, goals, skills, and/or roles.

It is a way of living a satisfying, hopeful, and contributing life even within the limitations caused by illness.

Recovery involves the development of new meaning and purpose in one's life as one grows beyond the catastrophic effects of mental illness (p. 15).

In the light of the context we have presented so far, we suggest that three basic assumptions in Anthony's definition should be re-considered.

Firstly, Anthony's definition describes recovery processes as *an individual process* without connections to the relational, cultural, material, and societal context around people.

Secondly, the definition describes recovery processes as merely *an internal psychological process*, as if recovery was only about changing one's cognitions of the world and of oneself in it, without any changes of people's living conditions.

Thirdly, the definition frames the recovery processes *within the boundaries of an illness model and even of chronicity*.

At the center of this assumption, we find a division between the (biological) illness and the (social) lived life of the person. Even when people develop a good life, the illness puts limitations on it. Living a good life does not have an impact on the illness. Paradoxically, chronicity is part of the vision, because even if people can grow beyond the effects of the illness, they cannot grow beyond the illness itself. This division might reflect the division between bio-medical treatment, psychotherapy and rehabilitation, where treatment is directed toward the illness and its symptoms, psychotherapy to the person's understanding of their situation and rehabilitation to the consequences of the illness in everyday life.

But the psychiatrization of recovery to an individual and mostly cognitive process may have contributed to the acceptance of Anthony's vision by international, national, and local agencies as it does not question the pillars of the dominant bio-medical model in “The decade of the brain “(1990–1999) and then “The century of the brain” (Mills, 2015; Karadzhov, 2021). If social and societal factors can cause and cure an “illness,” as the results from follow-up studies from the end of the twentieth century showed, then this illness might not be an illness, but a form of distress transformed/constructed as an illness.

The possibility of a total recovery includes the possibility to go not only beyond the consequences of the illness, but also beyond the illness itself. Total recovery opened the possibility to” become an ex” (Fuchs, 1988) and leave mental distress and services behind. On the contrary, Anthony's definition augment the number of persons judged to be in need of mental health services' interventions. The recognition of their specific experience-based knowledge opens for them careers as low-paid counselors in mental health structures with the obvious risk of adapting their knowledge to the dominant discourse, forming a kind of psychiatrized ≪sanctioned resistance≫ (Eriksson, 2015).

The life-long status of being in recovery reinforce the persons dependence of a psychiatric diagnosis to be able to receive different forms of social and economic support based on a recurring assessment of the individual need of the person. Thus, it hinders collective solutions to societal problems and creates users as agents of medicalization.

This is not to deny the hopeful message contained in the Anthony vision; that a decent and even good life is possible even when it comes to persons with a diagnosis of severe mental problems. But a close reading of his definition highlights that Anthony's a contextual understanding of recovery influences how we figure the relation between the lived life of people and their mental distress. It also influences our vision about how to support peoples' recovery journeys. In this way, the individualistic, cognitivistic, and illness limited understanding of recovery that has dominated the psy-field can be seen as a contribution to the psychiatrization of society.

The psychiatrization of our understanding of recovery reflects the diverse aspects (medicalization, individualization, bio-medicalization, pharmaceuticalization, and psychologization) included in the concept of psychiatrization mentioned in Mills (2015) and Beeker et al. (2021). The never-ending recovery process might be seen as old wine in new bottles, as it actualized mental distress as chronic and as illnesses. To live a fulfilling life becomes more a question of representations, personal will and capacity of adaptation than of actual resources in the form of social recovery capital (Tew, 2013).

## From Psychiatrization to De-Psychiatrization?—Are The Times Changing?

Previously we have stressed the importance of connections between societal changes and developments in the psy-field. Is it now possible to notice actual societal changes that could strengthen social perspectives and their applications to official mental health policies, practices, and the dominant understanding of mental distress?

### The Crisis of the Psychiatric Society

It is possible to argue that the actual neo-liberal period has led to growing inequalities and a growing proportion of persons who are not only marginalized but also excluded in high-income countries (Wacquant, 2009; Wilkinson and Pickett, 2018).

The dismantling of different sectors of the welfare state, such as reduction in school, health, financial and social support, together with the growing proportion of precariat on the work market have created an atmosphere of uncertainty and insecurity in large portions of the population in high-income countries (Castel, 1995; Peck, 2001). New Public Management as a way of organizing public services has led to a “proletarization” of mental health professions, reducing a growing part of their work to reproducing pre-determined interventions in narrowly defined evidence-based schema-bounded rituals (Pilgrim and McCranie, 2013).

In parallel to these societal developments, bio-medical psychiatry has strengthened its dominant position. However, at the same time, its shortcomings have become apparent (Rose, 2018). How can we explain the growing number of people given a psychiatric diagnosis? Frances (2013) wrote about an epidemic spread of diagnoses such as ADHD, depression and bipolar disorder. This epidemic can hardly be explained by a spreading of brain or genetic damages in the general population; instead, Frances points at the lack of scientific research behind the lowering of diagnostic criteria. Instead, he stresses the importance of the pressures of pharmacological companies (see also Brinkmann, 2016).

Despite repeated claims of being on the verge of discovering the biological bases of the major mental illnesses, bio-medical psychiatry has failed in this ambition (Priebe et al., 2013). This failure again came to light with the publication of the DSM-5 (Götzsche, 2013; Greenberg, 2013). Unable to give the ever-increasing number of diagnoses an acceptable reliability, bio-medical psychiatry still lacks a valid base for its practice (Imsel, 2013; Whitaker and Cosgrove, 2015; Johnstone et al., 2018).

Thus, we still lack studies showing statistical positive long-term effects of the major medicament interventions. As many studies are financed and controlled by the pharmacological companies, their results are in many cases biased and even then, are not so overwhelmingly positive (Moncrieff, 2009, 2013; Whitaker, 2010; Every-Palmer and Howick, 2014). Paradoxically, in the name of the Movement for Global Mental Health, pharmacological treatments and bio-medical classifications are introduced in low-income countries by the same WHO which has previously measured a greater possibility for recovery in these countries compared to bio-medicalized high-income countries (Mills, 2014).

### New Horizons?

The combined incapacity of the neo-liberal politicians and of bio-medical psychiatry proponents to live up to their own expectations creates a critical space where different forms of protests and counter actions have been actualized. The general societal dissatisfaction has produced a range of movements, many of them along the lines of the ones we saw in the 1970th, such as “Black Lives Matter,” and “Me Too,” but also more global critics such as the “Gilets Jaunes” and the struggle for societal changes to preserve the environment.

A trial with a guaranteed basic income was earlier implemented in Canada (Forget, 2019). There are now some attempts to introduce general basic income in Finland, Sweden, and Spain to secure a decent income for all citizens, irrespective of the cause of their need for support. A main shift in the development of guaranteed basic income is to transform a negative definition of people receiving support as “dependent” into being part in a process of societal solidarity. A user network, “Recovery in the bin,”^1^ formulates its fourth key principle of recovery as: “We want a robust ‘Social Model of Madness & Distress' building upon the Social Model of Disability and Independent Living, meaning support where needed and not perpetual pressure toward unattainable self-sufficiency” (https://recoveryinthebin.org).

In the psy-field the contextualization of mental health and recovery has not limited itself to research. Social and societal mental health practices have developed. The best known might be the one in Trieste where the mental health services work completely without inpatient structures. Mental health centers are spread in the community and offer different interventions and possibilities to join cooperatives and to participate in cultural activities (Scheper-Hugues and Lowell, 1986; Mezzina, 2006). Trieste has inspired services in different parts of the world, but refuses to be a model (Burns and Foot, 2020).

The Open Dialog Approach, developed in Finland and now applied in diverse parts of the world (Lakeman, 2014; Seikkula, 2019), emphasizes that help should start immediately and outside psychiatric structures. Through social network meetings the practitioners aim to include the diversity represented in the network. Tolerance of uncertainty is emphasized in order to make possible a multi-voiced, transdisciplinary collaboration involving those concerned by the situation (Holmesland et al., 2010). In a recent paper it is explicitly argued that Open Dialogue Approach may offer a less psychiatrizing form of support through its potential to (1) limit the use of neuroleptics, (2), reduce the incidences of mental health problems, and (3) decrease the use of psychiatric services (Von Peter et al., 2021).

“Recovery in the bin” and the Hearing Voices Network are other signs of growing alternative approaches to distress. Public medicine-free inpatient treatment is carried out some places in Norway (Cooper et al., 2021). Service users' right to influence their treatment and to participate in research about mental health has been recognized in official documents in different countries. One application of this has been the instauration of the possibility for mental health service users to decide about self-referral admission at their local inpatient clinic (Møller Olsø et al., 2016).

## The De-Psychiatrization of Recovery

During the last two decades, studies have been published about the role of contextual factors in mental health and recovery (Ware et al., 2007; Yanos et al., 2007; Read, 2010; Tew, 2013; Read et al., 2017; Ramon, 2018), about the shortcomings of a bio-medical understanding (Whitaker, 2010; Götzsche, 2013; Priebe et al., 2013; United Nations, 2017, 2021) resulting in a call for a paradigm shift from a bio-medical to a social paradigm (Priebe et al., 2013; Boyle and Johnstone, 2018).

A social and societal paradigm of the psy-field should include attention to aspects such as the construction of normality and deviance, and the transformation of deviance and distress into illnesses (Conrad and Schneider, 1992; Brinkmann, 2016; Rose, 2018). It should include the political decisions increasing inequalities causing distress (Priebe, 2016; Wilkinson and Pickett, 2018). It should also include the conditions for the development of social relationships and their impacts on people's sense of self (Davidson and Strauss, 1992; Schreiber, 1996; Sells et al., 2004). It should include the organization of support to people with mental distress, and finally it should include the social and societal context (Tew, 2013) and changes that impact on people's recovery processes (Boyle and Johnstone, 2018).

Social and societal factors should not be considered as mere triggers for an internal biological vulnerability. They are the basic conditions causing the development of mental distress (Read et al., 2009). Several contributions to a shift to a contextual understanding of distress and recovery in high-income countries have appeared recently. Just to mention a few:

The Power-Threat-Meaning framework defines behaviors and representations traditionally considered as symptoms of an illness as threat responses to abuse of power toward people in situations where they were fragile and could not mobilize enough resources to counter these threats (Boyle, 2020). Power imbalances tend to perpetuate themselves, deepening the distress and hindering changes in threat responses.

The concept of recovery capital (Tew, 2013) offers a way of mapping different aspects of core importance to initiating and maintaining a recovery journey. It is about economic (money at one's disposal), social (resources in one's social network), identity (relations with significant others), personal or mental (coping and ways of seeing oneself) and relationship capital (the quality of close relationships) at people's disposition and thus what kind of capital they might lack.

“Recovery in the bin” call themselves a critical theorist and activist collective. In one of their documents, they wrote:

We stand opposed to mental health services using “recovery” ideology as a means of masking greater coercion. We believe that this rise is a symptom of neoliberalism and that a meaningful “recovery” is impossible for many of us because of the intolerable social and economic conditions, such as poor housing, poverty, stigma, racism, sexism, unreasonable work expectations, and countless other barriers (https://recoveryinthebin.org).

Their main focus is on social inequalities and the risk that an individualizing understanding of recovery can become a tool for adaptation to a system producing distress.

### The Cause of All Causes

According to the Power-Threat-Meaning framework, poverty might be considered as “the cause of all causes” (Johnstone et al., 2018, p. 5) and could be a good starting point for a practical application of the reasoning above.

The connection between a person's economic status and mental health was established early (Hollingshead and Redlich, 1958; Eaton, 2001; Mills, 2015). Hansson et al. (1999) observed that even in welfare states, poverty was mentioned as one of the three top worries by service users, even prior to symptom relief. Poverty affects different aspects of life such as “nutrition, clothing, housing, education, traveling, participation in cultural, and leisure activities” (Ramon, 2018, p. 8). Thus, it is easy to understand that living a life of poverty constitutes a stress for oneself and one's social surroundings. This is a realistic outcome. But the consequences of poverty for the individual are often constructed as symptoms of an illness, a mental illness. Cohen (1993) pointed out similarities between what are considered as symptoms of mental illness and characteristics of poor people, such as depression, anxiety, and social isolation. Thus, the consequences of poor living conditions are transformed into illnesses based on an imbalance in the individual's brain. The person's economic state is one of the five recovery capitals mentioned by Tew (2013).

Social isolation might have different causes in different contexts, but it is assumed to be a characteristic symptom in persons with a diagnosis of severe mental illness. However, in a recent follow-up study of a general population, Mood and Jonsson (2015) showed a connection between increased poverty and a shrinking social network. They also noticed an opposite development in the same population as a consequence of an improved financial state. Changes in the size and composition of social networks in a general population can hardly be considered to be a result of sudden changes in people's brain functions. Wilton (2003) shows how poverty could hinder persons with severe mental problems from visiting their family even if they lived in the same town, as they could not afford public transportation. Brown (2015) and Topor et al. (2016a) mentioned how a decreased mutuality in social relations because of economic limitations could lead to a thinning and even ending of social relationships, a process also noted with people without “mental illness” (Offer, 2012).

Looking at contributing conditions to a recovery process, several studies (Davidson et al., 2001a,b; Sheridan et al., 2015; Topor et al., 2016a,b; Topor and Ljungqvist, 2017) have described how an unconditional improved financial situation was associated both with an improved social life, but also with improvement regarding symptoms, quality of life and functional level. Thus, social policy expressed in welfare state financial interventions should be able to prevent the development of distress and to contribute to a recovery process. Having a decent home to invite friends and family, and the possibility to offer a coffee or a gift recreates a sense of reciprocity central to the construction of a sense of self based on “living a satisfying, hopeful, and contributing life” as Anthony wrote. Improved finances might make it possible to create a home out of a housing (Borg et al., 2005) and may also create the conditions for the person to widen their enabling or therapeutic landscape, thus meeting new persons, having new experiences, and discovering new aspects of life in new settings (Duff, 2011; Doroud et al., 2018; Larsen et al., 2021).

It is a paradox that studies referred to earlier (WHO, 1979; Hopper et al., 2007) showed that more people recover in low-income countries compared to high-income countries with developed welfare states. Besides the hypotheses that were presented earlier in this article (concerning extended families, permeable labor market, spiritual understandings of mental distress, and limited medicalization), it might be possible to see the welfare state as created to overcome situations connected to industrialized societies, including the end of previous forms of solidarity, the appearance of long-term illnesses, harsher conditions on the labor market, and greater social isolation and thus increased fragility in the population. In this perspective, on the one hand, welfare states could be considered as expressions of solidarity between citizens, in contrast to the growing dominance of discourses about self-made individuals and their private responsibility for their own fate. Thus, recovery might be facilitated through the presence of a *general* welfare state palliating the effects of inequalities and of lacks in the person's recovery capital (Tew, 2013). On the other hand, welfare interventions based on *individual* assessments of needs (regarding economic support, support measures in school, etc.), might be necessary, but have been criticized as often being patronizing and normalizing and, as we mentioned above, have increasingly become based on diagnosis, another sign of the psychiatrization of society. New models have been developed to overcome these tendencies, such as “relational welfare” (Cottam, 2011) aimed at counteracting further bureaucratization of individual-based welfare administration through ≪Co-creation approaches, linked to a “new public governance” perspective≫ (Von Heimburg and Ness, 2021, p. 641).

Sociopolitical decisions might be of great importance for people's possibility to create a decent social life and a sense of self as an agent in one's life. Community centers, offering low-cost coffee, meals and activities, are highly valued (Estroff, 1985; Larsen and Topor, 2017), but risk becoming segregated and segregating places if the persons visiting them do not also have the possibility to go to coffee shops and other commercial and cultural places in the city. Deegan (2004) once said “Our needs are not special. Our needs are the same as your needs. (…) We don't want what you are giving; we want what you have got” (p. 11).

### A New Vision

The social is personal. This is the case both regarding the emergence of mental distress and recovery from mental problems (Mezzina et al., 2006; Topor et al., 2011; Tew et al., 2012; Rose, 2014; Boyle and Johnstone, 2018). Different research traditions have developed looking at peoples' recovery in context such as enabling places (Duff, 2012), post asylum landscapes (Högström, 2018), assemblage (Larsen et al., 2021), and relational (Price-Robertson, 2017). The centrality of civil rights and of social injustice has been stressed in different studies (Harper, 2020; Reis et al., 2022; Zeira, 2022).

In the light of both earlier and current research findings we think it would be justifiable and possible to update Anthony's widespread definition of recovery; as an anti-thesis to its psychiatrization.

As research has shown, recovery happens all the time in the most different situations. There cannot be one recovery method or one recovery plan that fits all, but, from above mentioned research, we know a lot about conditions distressing persons and hindering or facilitating the start and sustainability of processes of recovery in the global North (Priebe, 2015, 2016). Recovery is as much a question of social and material changes as it is of personal development; therefore, a tentative definition should situate the personal aspects within social aspects such as social relationships and living conditions:

Recovery is a deeply social, unique, and shared process in which our living conditions, material surroundings, social relations and sense of self evolve.

It is about striving to live satisfying, hopeful, and reciprocal lives, even though we may still experience threats, stressful social situations, and distress.

Recovery involves engaging in encounters and dialogs where new ways of understanding and handling one's situation are created as we move beyond the psycho-social-material crisis.

The psychiatrization of society is a main hinder to recovery as it transforms distress based on social injustice and power imbalance into individual illness. The psychiatrized society demands diagnosis and medical treatment as a condition for economic and social support. However, we can see signs of resistance and the development of alternatives to this psychiatrized “guiding vision.” Redefining recovery and recognizing the importance of social, material, cultural and relational aspects involved in recovery processes and thus behind mental distress is part of the challenge of de-psychiatrizing society.

## Author Contributions

AT, TDB, and IBL contributed to the formulation of the background and aim of the study. AT wrote a first draft of the article that was discussed on several occasions by all the authors. A second draft written by AT was then discussed and supplemented by TB and IL. All authors contributed to manuscript revision, read, and approved the submitted version.

## Funding

The open access fee has been received by the University of Agder (Norway).

## Conflict of Interest

The authors declare that the research was conducted in the absence of any commercial or financial relationships that could be construed as a potential conflict of interest.

## Publisher's Note

All claims expressed in this article are solely those of the authors and do not necessarily represent those of their affiliated organizations, or those of the publisher, the editors and the reviewers. Any product that may be evaluated in this article, or claim that may be made by its manufacturer, is not guaranteed or endorsed by the publisher.

## Footnotes

^1^Recovery in the bin: https://recoveryinthebin.org.
